# More GC Means More RNA

**DOI:** 10.1371/journal.pbio.0040206

**Published:** 2006-05-23

**Authors:** Richard Robinson

The genetic code that dictates the translation of RNA triplets into amino acids is rife with synonyms. Both CUG and CUA encode the amino acid leucine, for instance, and CCC and CCU encode phenylalanine. Each member of these synonymous pairs works equally well—the use of any particular synonym has no effect on the incorporation of its corresponding amino acid. But in mammalian genes, there is a surprising cumulative effect—genes with a greater proportion of third-position Gs or Cs are expressed more than genes with third-position As or Us. This effect is so pronounced that some researchers use the GC content of a multigene segment of a chromosome as a rough measure of its protein-producing activity.

There is a long path from gene to protein, and it is unclear at what step along that path the GC versus AU difference exerts its influence. A new study by Grzegorz Kudla, Leszek Lipinski, and colleagues show that the difference is neither in translation of RNA into protein nor in RNA stability, but in production, or transcription, of RNA from the DNA template.

The authors began by comparing two genes that encode protein-stabilizing heat shock proteins, Hsp70 and Hsc70. The coding regions of the two genes are similar in length, and the proteins are largely similar in sequence. The “GC3” for Hsp70 is 92%, meaning it uses a G or C at 92% of its third positions, while the GC3 for Hsc70 is 46%. Within the cell, however, the genes also differ in their location in the chromosome, their promoters (which bind the RNA-synthesizing machinery), and other important determinants of gene expression. To control for these potentially confounding factors, the authors introduced the genes for Hsp70 and Hsc70 into cells in chromosome-independent vectors. They found the GC-rich gene produced ten times as much protein as the GC-poor one, an effect that was independent of the type of cell. This effect was not due to the rate of protein synthesis, which was the same when equal amounts of the messenger RNA for each gene was present. Neither was it due to differences in RNA stability, which was also similar for the two genes. Instead, the difference was due to production of RNA.

This effect was not limited to the heat shock proteins. Similar differences were seen with GC-rich and -poor versions of genes for the mammalian immune system protein interleukin-2, as well as for green fluorescent protein, from the very nonmammalian jellyfish. Neither was it limited to the extrachromosomal position of the introduced gene: GC-rich genes integrated into the chromosome outperformed GC-poor ones in exactly the same spot.

This study does not address the “how” of the GC advantage—the mechanism by which GC-rich genes are transcribed more than GC-poor ones. But it does identify the “when,” which should speed research into the mechanism, and may ultimately help illuminate the “why”—the evolutionary reasons behind this difference. These results also confirm the utility of increasing GC content of genes used for medical therapies, where increased expression can mean the difference between life and death, and for biotechnology applications, where more protein means more product and more money.

## 

**Figure:**
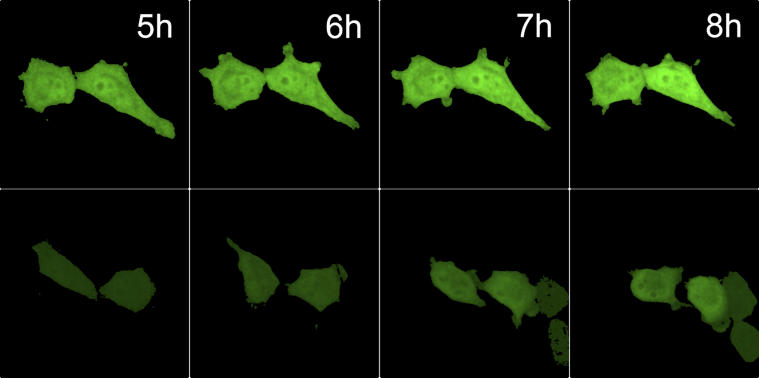
Cultured human cells produce ten times more green fluorescent protein from a GC-rich gene (top row) than from a synonymous GC-poor gene (bottom row).

